# Effect of group impromptu music therapy on improving test anxiety and emotional regulation ability in medical students

**DOI:** 10.3389/fpsyg.2024.1467830

**Published:** 2024-12-12

**Authors:** Li Song, Rong Xiao, Changjing Wang, Chaoyang Li, Qi Liu, Ying Zhang, Zhen Liu, Lei Zhang, Ming Zhang

**Affiliations:** ^1^Qingdao Medical College, Qingdao University, Qingdao, China,; ^2^Normal College of Qingdao University, Qingdao University, Qingdao, China,; ^3^The Affiliated Hospital of Qingdao University, Institute for Translational Medicine, Qingdao University, Qingdao, China,

**Keywords:** test anxiety, emotional regulation, group impromptu music therapy, medical students, improvement

## Abstract

**Introduction:**

Test anxiety, a prevalent psychological issue among medical students, can profoundly impact their social, behavioral, and emotional development. This condition is significantly associated with challenges in emotional regulation, and to date, no effective clinical treatment has been established to address it. This study aimed to investigate the potential benefits and effects of group impromptu music therapy (GIMT) on enhancing emotional regulation skills and alleviating test anxiety in medical students.

**Methods:**

A total of 29 participants in the experimental group and 30 participants in the control group were finally included for data analysis. Four weeks prior to the final exams, the experimental group commenced GIMT treatment, while the control group did not receive any intervention. Following the four-week intervention period, both groups were assessed using standardized scales and follow-up semi-structured interviews.

**Results:**

The results revealed that students given the GIMT intervention reported significantly lower levels of test anxiety, as measured by the Test Anxiety Scale (TAS), and experienced fewer difficulties with emotional regulation, as indicated by the Difficulties in Emotion Regulation Scale (DERS). Additionally, these students achieved higher examination scores than the control group. Qualitative data from semi-structured interviews further supported these observations.

**Discussion:**

Collectively, these findings demonstrate that GIMT is an effective method for enhancing the emotional regulation abilities of medical students and for relieving the symptoms of test anxiety, ultimately resulting in better academic performance. The study also indicates that GIMT could be a promising clinical auxiliary method for dealing with test anxiety and should be considered for inclusion in the curriculum of healthcare professional education programs. Nonetheless, more efforts are needed to address the limitations of this method before it can be widely used for clinical practice.

## Introduction

Anxiety is an emotional state characterized by tension, worry and/or fear regarding future or ongoing events. Excessive anxiety can evoke a range of negative emotions, such as fear, stress, helplessness and anger (Putwain et al., 2021), and may even lead to emotional disorders and psychological issues. “Test anxiety” specifically refers to the psychological and behavioral responses that individuals experience when they are worried about possible failure in exam or comparable evaluative situation (Bodas and Ollendick, 2005; von der Embse et al., 2018). Test anxiety is a widespread issue globally (Yusoff et al., 2013; Kulsoom and Afsar, 2015; Nazir et al., 2021), affecting up to 40% of students (Neil and Christensen, 2009).

In China, medical students are particularly prone to severe test anxiety (Mao et al., 2019; Zeng et al., 2019; Li et al., 2022; Shi et al., 2022). They experience higher levels of stress than their peers in other disciplines (Shao et al., 2020). Excessive test anxiety can have detrimental effects on learning, academic achievement, and psychological well-being. The cognitive interference and worry caused by test anxiety before exams may lead to poor understanding of study material and impair effective study habits, ultimately undermining test performance (Chapell et al., 2005; Jerrim, 2023). Therefore, an inverse relationship has been observed between test anxiety and grade point average (GPA) (Chapell et al., 2005; Jerrim, 2023). Additionally, studies has noted gender disparities in the levels of test anxiety and the impact of test anxiety on academic performance (Bolbolian et al., 2021; Dowker and Sheridan, 2022).

Emotional regulation is defined as the process through which individuals modulate their emotions, determining when they occur, and how they are experienced, and how they are expressed (McRae and Gross, 2020). Studies have shown that effective emotional regulation can reduce anxiety levels and promote psychological resilience (Aldao et al., 2010; Moltrecht et al., 2021). A recent study has revealed a significantly inverse correlation between emotional regulation and test anxiety among medical students (Liu et al., 2021). This finding suggests that a strong ability to regulate emotions may mitigate test anxiety, while poor emotional regulation might aggravate the symptoms of test anxiety. Therefore, strategies aimed at improving emotional regulation are likely to be effective in alleviating the adverse reactions of test anxiety.

Medical interventions can reduce anxiety (Baldwin et al., 2005; Moltrecht et al., 2021), but these treatments may come with other side effects, including gastrointestinal discomfort, jitteriness, insomnia, and headache (Hodo, 2006; Garakani et al., 2020). Cognitive behavioral therapy (CBT) is widely recognized as a first-line psychotherapy for anxiety (Fachner et al., 2013; Lim et al., 2015; Bandelow et al., 2022). However, certain students, especially those under significant emotional regulation pressure, may have difficulty expressing their thoughts and emotions verbally (Zhang et al., 2022). This highlights the limitations of current treatment strategies for test anxiety, necessitating the development of more diverse and innovative prevention methods.

Art therapy is an important component of the realm of therapeutic treatment and has been used in various rehabilitation and recovery processes. In the field of mental health, modalities such as music therapy (MT), painting, and craft therapy are increasingly being recognized for their potential benefits. MT, in particular, uses clinical and evidence-based music interventions to achieve the personalized goals set by qualified practitioners (de Witte et al., 2022). It is distinct from music medicine interventions, which are mostly administered by medical or healthcare professionals (Dileo, 2006). An increasing number of clinical trials have substantiated the efficacy of MT in alleviating test anxiety (Eyüboğlu et al., 2021; Galal et al., 2021). Consequently, MT has been taken as an alternative or complementary treatment for psychiatric disorders, including test anxiety (de Witte et al., 2020; Lu et al., 2021; de Witte et al., 2022).

Group impromptu music therapy (GIMT) is a specific approach of MT. By engaging in collective musical improvisation with various instruments, music therapists facilitate an environment where participants can release negative emotions, establish interpersonal connections, articulate their inner experiences, and accept others through safe and nonverbal “quasi-socialized” interactions. Such activities are conducive to the improvement of emotional regulation and psychological adaptability (Sandak et al., 2019). Our previous research has demonstrated that GIMT can effectively enhance emotional regulation and reduce depressive symptoms among college students (Zhang et al., 2022). However, the potential efficacy of GIMT in reducing test anxiety remains unknown.

Studies have explored different ways to mitigate test anxiety, such as the use of placebo pills, yogic breathing techniques, animal-assisted therapy, online laughter therapy sessions, hand massage, etc. (Quinn and Peters, 2017; Kaur Khaira et al., 2023; McCormick and Lamberson, 2024; Jirjees et al., 2024). Our study introduced GIMT for the first time to address test anxiety. Through the processes of empathy, mirroring, dialogue, emotional exploration and discussion, we anticipated participants would experience positive transformations during GIMT. This paper aimed to investigate the effects of GIMT on test anxiety and emotional regulation in medical students. We adopted a randomized controlled trial design to administer GIMT. Furthermore, we integrated quantitative data from assessment scales with qualitative insights derived from semi-structured interviews to provide a comprehensive evaluation of the therapy’s effect. We hypothesize that GIMT can effectively alleviate test anxiety symptoms in medical students by improving their emotional regulation skills, which could consequently lead to better academic performance.

## Materials and methods

### Participants

From October 2022 to December 2022, the project team conducted recruitment and testing. The age range of 18 to 25 years is commonly designated as “emerging adulthood” rather than adolescence (Hochberg and Konner, 2019). Individuals in their early twenties no longer identify themselves exclusively as teenagers, but they do not fully embody the characteristics of mature adulthood. This transitional period has been recognized as a distinct developmental phase in human life characterized by intricate transformations in neurobiology, psychology, and social systems (Gomes et al., 2019). Such changes can precipitate instability, potentially triggering anxiety attacks or worsening symptoms in vulnerable individuals (Luo et al., 2024). In light of these considerations, our study focused on students around the age of 20, who are in the “emerging adulthood.” Finally, 250 students from Qingdao University participated in the initial testing session.

The selection methods and criteria are: (a) undergraduate medical students who will have an exam at the end of the semester; (b) 18 ~ 22 years old; (c) Individuals who score ≥ 101 on the Difficulties in Emotion Regulation Scale (DERS) and ≥ 20 on the Test Anxiety Scale (TAS).

The exclusion criteria are: (a) past or present mental illness; (b) current engagement in MT or other anti-anxiety treatments elsewhere; (c) the presence of acute suicidal thoughts or behaviors; (d) the existence of organic diseases. The criteria for withdrawal from the study are: (a) non-participation in more than two consecutive music therapy sessions; (b) failure to attend the final assessment; (c) a voluntary request to withdraw from the study.

After selection, 64 students who scored ≥101 on the DERS and ≥ 20 on the TAS, and exhibited test anxiety symptoms, were assigned to either a control group (*n* = 32) and or an experimental group (*n* = 32) using a simple randomization method. The simple randomization was performed by a research assistant not involved in the study, using the Research Randomizer.^1^ A card with a randomly assigned numbers designating the groups were placed inside opaque envelopes, and shuffled. As each participant arrived, the research assistant opened an envelope in sequence to reveal the group assignment.

**Figure 1:**
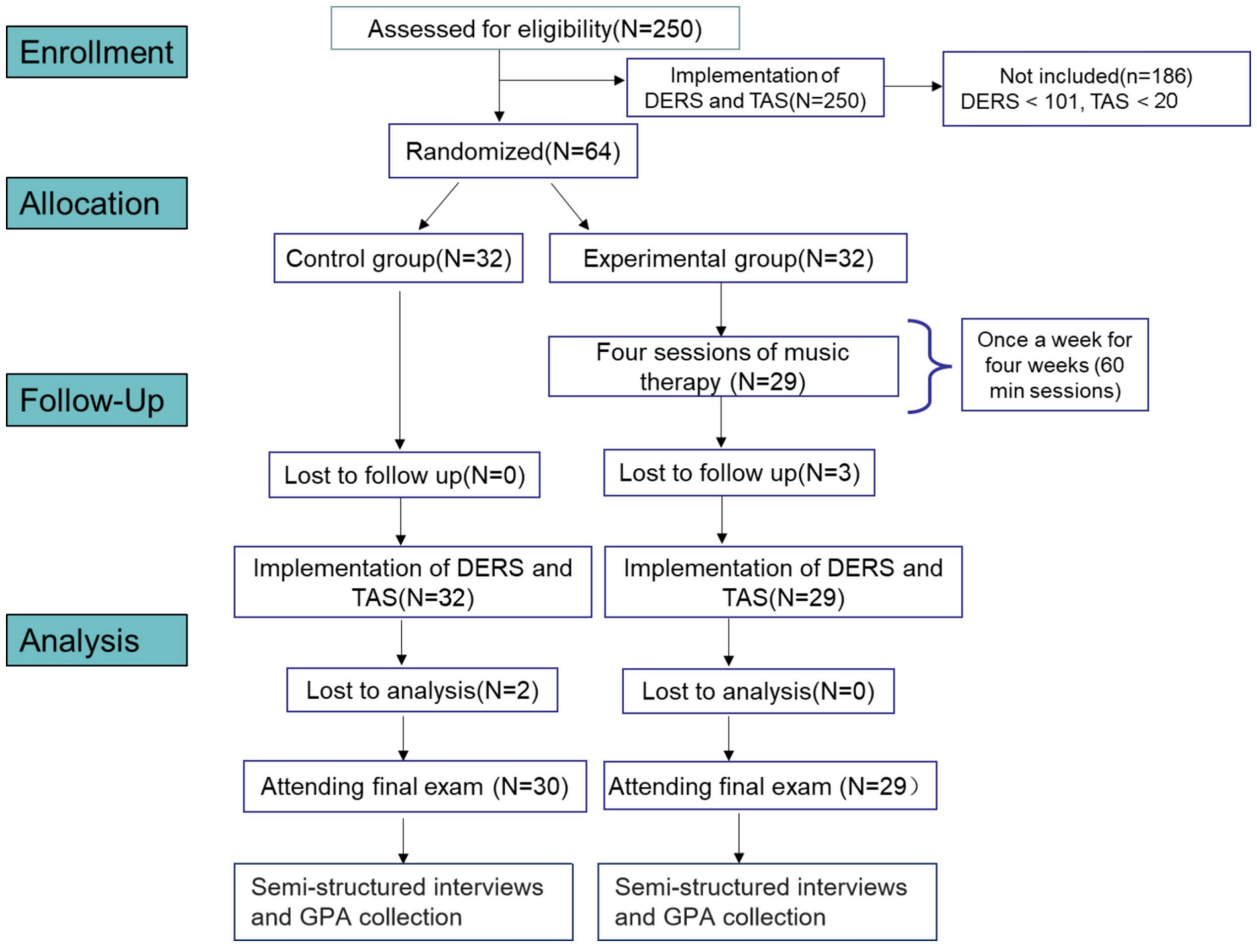
The participants and data collection procedures of the GIMT intervention. 250 volunteers were assessed and then 64 participants wer enrolled.They were randomly divided into two groups. After the GIMT intervention, 30 participants in the control group and 29 participants in the experimental group were assessed by the DERS, the TAS and semi-structured interviews.

Post-randomization, we assessed whether any participants had close friends in the opposite group with whom they usually shared emotions. To prevent bias and ensure that no participants had familiar individuals in both groups, those with intimate relationships across the two groups were excluded. Additionally, three students were excluded from the experimental group for missing more than two MT sessions, and two students who failed to complete the data collection forms were excluded from the control group during the analysis period. Ultimately, the research was concluded with 59 students: 19 females and 11 males in the control group, and 19 females and 10 males in the experimental group (Figure 1).

All participants were informed of the aim and method of the study, and their written informed consents were securely obtained. The whole protocol of this study was admitted by the ethics committee of the medical college of Qingdao University.

### Scales

The DERS is an important tool for assessing individual capacities for emotional regulation. Comprising a 5-point Likert scale across 36 items, the DERS is structured into six dimensions: emotional perception, emotional understanding, emotional response acceptance, emotional impulse control, goal-directed behavior stimulation, and effective use of emotional regulation strategies (McVey et al., 2022). The higher the score, the worse the ability of emotional regulation (McVey et al., 2022). An adapted Chinese version of the DERS developed by Li et al. (2018) was used in this study. The Cronbach alpha coefficient of the DERS in our study was 0.762, indicating the scale’s reliability.

The TAS was developed based on the Sarason’s Test Anxiety Scale by Wang (2001). The TAS is designed to explore the frequency with which the students experience anxiety before, during, and after taking tests. The TAS is a true-false, 37-item scale. A high total score indicates a higher level of test anxiety (maximum score 37). Respondents are divided into three levels according to TAS scores: points ≥ 12, low test anxiety level; 12 < points < 20, normal anxiety level; points ≥ 20, high test anxiety level. In our study, the Cronbach alpha coefficient of the TAS was 0.868, which confirms the reliability of the TAS.

### GIMT intervention

The project director and intervention implementers are certified and registered music therapists. They are supported by two assistants, one male and one female, who are certified psychological counselors from the school’s mental health education center. These assistants are mainly responsible for recording the emotions and language expressions of the participants during the activities, as well as assessing the team’s status, and providing timely summaries and feedback. The project director has undergone GIMT system training and did not participate in the GIMT intervention to avoid bias. They all have over 7 years of experience in psychological counseling.

The GIMT intervention was strategically scheduled to occur 4 weeks before the exam week. It was implemented at the Mental Health Education Center of Qingdao University. The intervention was structured as a weekly session, held every Tuesday for a duration of 60 min each time. In the GIMT sessions, the students in the experimental group were treated as a collective, working together under the guidance and promotion of music therapists. All participants freely play percussion instruments with low technical thresholds such as drums and jointly create personalized music, ensuring that all participants could easily join in, regardless of their musical background. This method is inclusive and can focus on the emotional experiences rather than technical proficiency. Freely playing instruments and creating personalized music provide outlets for participants’ creativity and expression. During this process, the therapists synchronously tracked the students’ performance. This allowed them to stimulate and amplify the students’ emotional experience and foster a sense of emotional resonance among participants. Following each impromptu performance, the therapists engaged in discussions with the students about their emotional experience triggered by the performance. This reflective step of the intervention is critical for helping participants understand their emotions, which is a key aspect of emotional regulation.

The GIMT intervention was exclusively implemented for the experimental group, Meanwhile, during this same period, the control group continued with their regular routines of living and studying without any GIMT-related interventions.

### Data collection

Students with total scores ≥101 on the DERS and ≥ 20 on the TAS were individually interviewed. After GIMT period, the students in both groups underwent the DERS and TAS tests once more. Subsequently, both groups were followed up for 4 weeks. Throughout the follow-up, the students’ examination grades were collected and recorded for analysis.

Semi-structured interviews were employed to qualitatively analyze the intervention effect by two certified psychological counselors (Badrian et al., 2022; Kallio et al., 2016). The interviews took place in a quiet room at the Qingdao University. There was not anyone else present besides the participants and researchers to ensure privacy and focus. The interviews were scheduled to occur after GIMT implementation. First, the interview began with a series of open-ended questions (Table 1). For example: “How do you feel after a period of the GIMT intervention?” Subsequently, the inquiry could proceed with questions like, “Do you perceive any changes in your test anxiety level?” Exploratory questions were also used during the interviews to delve deeper, such as, “Could you explain more?.” The interviews continued until data saturation. There were no repeat interviews. The average duration of each interview was approximately 65 min. Students shared their emotional states and psychological experiences after the GIMT intervention openly. Related information was recorded and summarized by two counselors. In particular, as compensation, the control group also received GIMT intervention after all testing were completed without follow-up evaluations.

**Table 1:** Interview guide.

Interview time	Example semi-structured interview guide	Probing question
After GIMT intervention	How do you feel after a period of the GIMT intervention?	Could you explain more?
	Do you perceive any changes in your test anxiety level?	What aspects of anxiety have been relieved or disappeared?
	Can you give an example of feeling relieved of anxiety during GIMT?	Could you explain more?
	What musical elements impress you more in GIMT? How do you think they affect your emotions?	Rhythms, melodies, harmonies?
	Do you feel more motivated to review and study?	Could you explain more?
	Has the efficiency of learning changed?	Could you explain more?
	What is your current understanding of exams?	Please give me more information.
	Do you think this music therapy can raise your confidence in dealing with future exams?	How did it help?

### Trustworthiness

In consideration of the trustworthiness, we referred to the criteria developed by Guba and Lincholn (1994), which encompass credibility, dependability, confirmability, and transferability. We spent sufficient time carefully collecting data and investigating participants, with two researchers consistently followed up on the data collection process. Throughout the data collection process, researchers did not mention any research hypotheses and conduct any purposeful analysis. The conventional content analysis approach was applied for data analysis. Our research results were reviewed by participants and experts for approval and feedback. Researchers meticulously reviewed the criteria, and discussed the open coding till all problems were satisfactorily resolved.

### Data processing

The data sets of each scale were analyzed by SPSS version 23.0 software. The normality distributions of all quantitative data were checked using the Shapiro–Wilk normality test. All data sets were shown to be normally distributed (*p* > 0.05). Therefore, independent *t*-test was used to assess differences between different groups. Paired samples *t*-test was employed for within-group comparisons of pre- and post-intervention outcomes. The data were represented as mean ± standard deviation (SD). The difference of *p* < 0.05 was considered statistically significant.

### Data analysis

The characteristics of the experimental and control subjects, including age, height, weight, GPAs, TAS scores and DERS scores, were compared using independent *t*-test to determine whether the effectiveness of the randomization process. Then, the effects of the GIMT intervention on test anxiety and emotional regulation were explored quantitatively by conducting paired samples *t*-tests to compare the TAS or DERS scores of the same individuals before and after the GIMT intervention. In the follow-up process, semi-structured interviews were carried out to qualitatively analysis the effects of the GIMT intervention. Moreover, GPAs of the experimental and control groups were compared using independent *t*-test to indirectly reflect the influence of GIMT on test anxiety.

## Results

### Characterization of the experimental and control groups

We analyzed the characteristics data of participants using independent *t*-test. Results showed that there were no significant differences between the control group and the experimental group in terms of age (control group:19.83 ± 1.05 years; experimental group: 19.72 ± 1.07 years. *p* = 0.92 > 0.05), height (control group:169.07 ± 7.52 cm; experimental group: 168.36 ± 8.13 cm. *p* = 0.74 > 0.05), and weight (control group: 59.28 ± 10.76 kg; experimental group: 59.98 ± 13.87 kg. *p* = 0.21 > 0.05). Additionally, 63.3% of the control group (19/30) and 65.5% of the experimental group (19/29) were female, indicating no significant difference in the gender composition of the two groups (*p* = 0.86 > 0.05). Moreover, the two groups had similar GPA from the last semester (control group: 2.22 ± 0.65, experimental group: 2.20 ± 0.68, *p* = 0.78 > 0.05). All studies have confirmed the similarity between the two groups, indicating that they can be used for subsequent grouping experiments.

**Figure 2:**
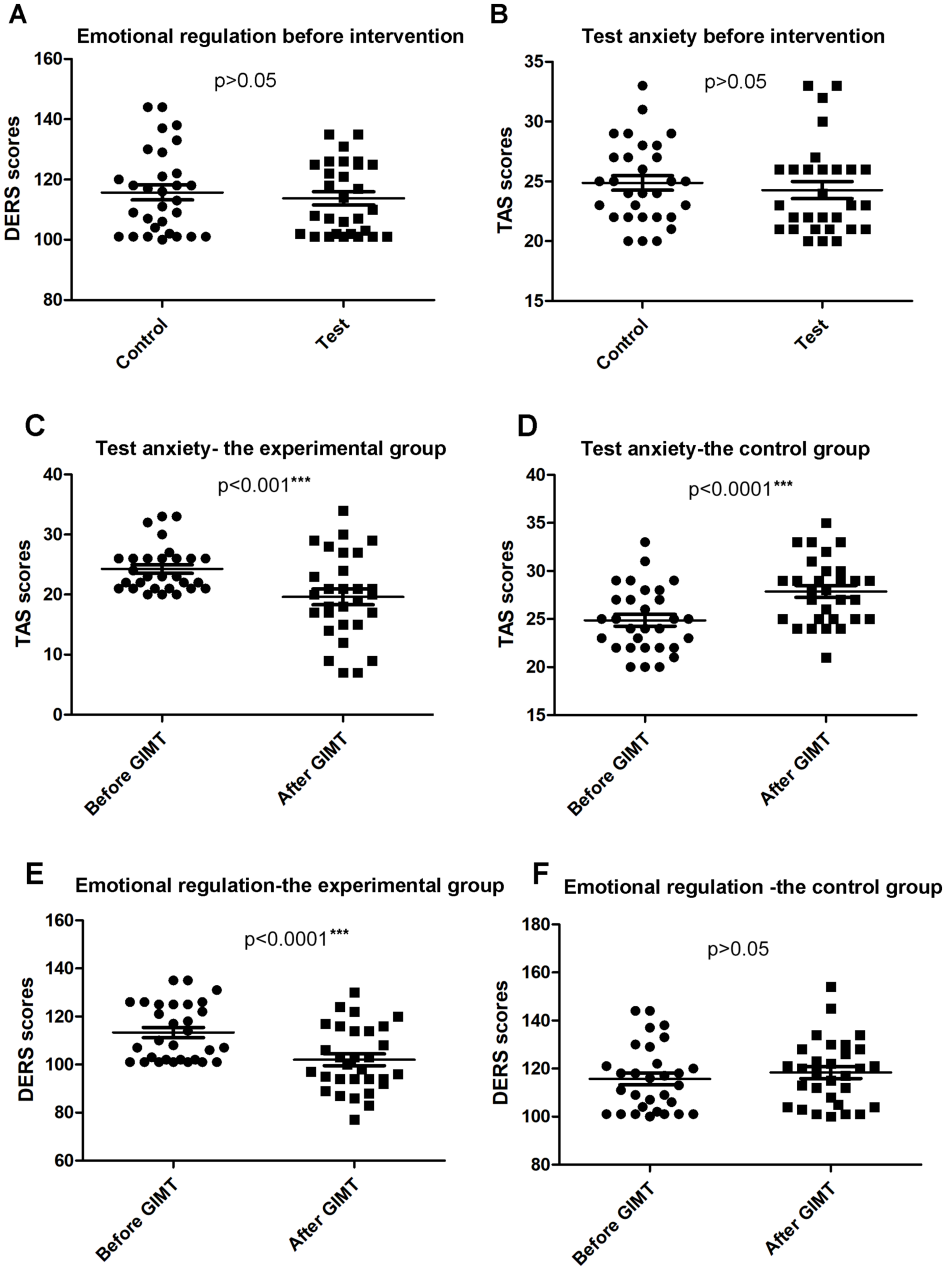
Analysis of the TAS and DERS data for the experimental group and the control group. **(A)** There were no significant differences between the control and experimental groups in emotional regulation before intervention. **(B)** There were no obvious differences between the control and experimental groups in test anxiety before intervention. **(C)** After intervention, the TAS score was reduced in the experimental group. **(D)** After intervention, the TAS score was increased in the control group. **(E)** After intervention, the DERS score of the experimental group was reduced. **(F)** The DERS score of the control group shows no difference before and after the GMIT intervention.

Regarding psychological assessment, data from the DERS and the TAS were statistically analyzed. Before the GIMT intervention, no significant differences were observed between the control group and the experimental group in DERS scores (control group: 115.73 ± 13.76; experimental group: 113.76 ± 11.78. *p* = 0.68 > 0.05) (Figure 2A) and TAS scores (control group: 24.87 ± 3.37; experimental group: 24.28 ± 3.86. *p* = 0.43 > 0.05) (Figure 2B). For the six dimensions of the DERS, there were also no obvious differences between the control group and the experimental group (*p* > 0.05) (Table 2).

**Table 2:** Distribution of DERS dimensions in the experimental and control groups before GIMT.

Dimensions	Experimental group	Control group	*p*	*t*	95% CI
Emotional perception	22.48 ± 3.47	23.30 ± 3.62	>0.05	0.89	−1.034 to 2.668
Emotional response acceptance	18.34 ± 3.30	18.40 ± 4.26	>0.05	0.06	−1.934 to 2.044
Emotional understanding	15.28 ± 1.39	15.37 ± 1.80	>0.05	0.22	−0.746 to 0.928
Goal-directed behavior stimulation	17.76 ± 2.49	18.00 ± 2.69	>0.05	0.36	−1.111 to 1.594
Effective use of regulatory strategies	23.76 ± 5.50	23.50 ± 4.96	>0.05	0.19	−2.986 to 2.468
Emotional impulse control	16.14 ± 3.45	17.17 ± 4.13	>0.05	1.04	−0.958 to 3.016

Independent *t*-test was used to assess differences between different groups. CI, confidence interval. All data were obtained from the corresponding test scales and expressed as mean ± SD.

### The effect of the GIMT intervention on test anxiety

TAS scores of both groups before and after the GIMT intervention were calculated by paired samples *t*-test. After GIMT, the TAS score of the experimental group was significantly decreased (before-test 24.28 ± 3.86, after-test 19.62 ± 7.10. *p* < 0.001***) (Figure 2C), suggesting a positive impact of the GIMT intervention on reducing anxiety levels. On the contrary, in the control group without intervention, the TAS score was remarkably higher (before-test 24.87 ± 3.37, after-test 27.87 ± 3.36. *p* < 0.0001***) (Figure 2D), implying that without intervention, anxiety levels in the control group may have worsened over time.

Considering gender factors, we classified the GIMT intervention results of boys and girls using independent *t*-test (Table 3). Before the GIMT intervention, there were no significant differences in TAS scores between boys and girls in both the control and experimental groups (*p* > 0.05), implying that anxiety levels were comparable between genders within each group. After the GIMT intervention, the results continued to show no obvious differences in TAS scores between boys and girls in each group (*p* > 0.05). These observations indicate that gender does not appear to be a factor influencing the GIMT intervention effect on test anxiety.

**Table 3:** Gender differences of GIMT effect in two groups in test anxiety and emotional regulation.

Scales	Groups	Before intervention					After intervention				
		Male	Female	*p*	*t*	95% CI	Male	Female	p	t	95% CI
TAS	Experimental group	23.90 ± 3.00	24.47 ± 4.31	>0.05	0.37	−3.719 to 2.571	17.60 ± 8.95	20.68 ± 5.90	>0.05	1.12	−8.749 to 2.581
17.807	Control group	24.72 ± 3.23	24.95 ± 3.54	>0.05	0.17	−2.881 to 2.441	27.54 ± 3.50	28.05 ± 3.36	>0.05	0.39	−3.154 to 2.140
DERS	Experimental group	112.10 ± 12.91	114.63 ± 11.41	>0.05	0.54	−12.093 to 7.030	99.10 ± 14.51	102.58 ± 12.82	>0.05	0.66	−14.229 to 7.271
	Control group	120.36 ± 13.65	113.05 ± 13.45	>0.05	1.43	−3.185 to 17.807	125.18 ± 15.22	114.37 ± 11.07	>0.05	2.25	0.951 to 20.676

All data were obtained from the corresponding test scales and expressed as mean ± SD. Independent *t*-test was used to assess differences between different groups. CI, confidence interval.

### The effect of the GIMT intervention on emotional regulation

DERS scores of both groups before and after the GIMT intervention were evaluated. There was a significant decrease in DERS scores after the GIMT intervention in the experimental group (before-test 113.76 ± 11.78, after-test 101.38 ± 13.28. *p* < 0.0001***) (Figure 2E), whereas there was no obvious alteration in DERS scores in the control group (before-test 115.73 ± 13.76, after-test 118.33 ± 13.57. *p* > 0.05) (Figure 2F), suggesting that the GIMT intervention was effective in improving emotional regulation.

Besides, we took into account the effect of gender on the effectiveness of the GIMT intervention on DERS scores (Table 3). According to Table 3, no significant changes were found in DERS scores between males and females in both the control and experimental groups post-intervention (*p* > 0.05), indicating that gender does not influence the impact of the GIMT intervention on emotional regulation.

After the GIMT intervention, the experimental group showed changed levels in six dimensions of the DERS. The scores of emotional perception, emotional response acceptance, emotional understanding, goal-directed behavior stimulation, effective use of regulatory strategies, and emotional impulse control of the experimental group were all significantly decreased compared with their pre-GIMT scores (Table 4). This decrease in the DERS scores suggests an improvement in emotional regulation ability. For the control group without the GIMT intervention, there were no significant differences in the scores of the six dimensions of the DERS. These findings indicate that the GIMT intervention is beneficial for enhancing emotional regulation abilities (Table 4).

**Table 4:** Scores of six dimensions of emotional regulation in the experimental and control groups.

Dimensions	Groups	Before GIMT	After GIMT	*p*	*t*	95% CI	Pairing effectiveness	
							*r*	*P*
Emotional perception	Experimental group	22.48 ± 3.47	20.45 ± 3.77	**	1.90	−1.943 to 0.07584	0.73	***
	Control group	23.30 ± 3.62	24.23 ± 3.71	>0.05	3.47	0.834 to 3.235	0.62	***
Emotional response acceptance	Experimental group	18.34 ± 3.30	15.21 ± 3.78	***	5.64	1.998 to 4.278	0.65	***
	Control group	18.40 ± 4.26	19.07 ± 4.00	>0.05	1.12	−1.885 to 0.551	0.69	***
Emotional understanding	Experimental group	15.28 ± 1.39	14.41 ± 1.72	*	2.72	0.213 to 1.511	0.41	*
	Control group	15.37 ± 1.80	15.80 ± 1.94	>0.05	1.37	−1.081 to 0.2147	0.57	***
Goal-directed behavior stimulation	Experimental group	17.76 ± 2.49	15.69 ± 2.80	***	5.34	1.276 to 2.862	0.70	***
	Control group	18.00 ± 2.69	17.87 ± 2.40	>0.05	0.31	−0.748 to 1.014	0.58	***
Effective use of regulatory strategies	Experimental group	23.76 ± 5.50	21.10 ± 4.75	***	4.02	1.302 to 4.008	0.77	***
	Control group	23.50 ± 4.96	24.13 ± 5.46	>0.05	0.91	−2.052 to 0.786	0.74	***
Emotional impulse control	Experimental group	16.14 ± 3.45	14.52 ± 3.41	**	0.31	0.543 to 2.698	0.66	***
	Control group	17.17 ± 4.13	17.23 ± 3.98	>0.05	0.39	−0.419 to 0.286	0.97	***

All data were obtained from the corresponding test scales and expressed as mean ± SD. The paired samples *t*-test was used for pre- and post-intervention comparisons of the same group. CI, confidence interval. *r*: correlation coefficient. * represents *p* < 0.05; ** represents *p* < 0.01; ***represents *p* < 0.001.

### Follow-up analyses of subjects’ emotional regulation and test anxiety

The follow-up analyses showed that the students in the experimental group experienced improved test anxiety levels after the GIMT intervention. The enhancements were evident in the following aspects: (a) The physical symptoms of test anxiety were attenuated. For instance, a participant commented, “I used to suffer from obvious gastrointestinal issues before exams. But after receiving GIMT, the occurrence of these symptoms has significantly decreased.” (b) They reported enhanced concentration and memory, as well as improved study efficiency. Most participants stated that they no longer felt difficult in concentrating on learning and their learning efficiency has increased. (c) Their self-confidence and self-esteem have increased. They tried to avoid over focusing on test results and exaggerating negative effects of poor results. Some participants reported a significant reduction in negative behaviors and they felt confident in facing exams. (d) There was a decrease in the frequency of negative self-talk and feelings of despair. They no longer avoid testing by skipping class or even considering dropping out of school. As one participant put it, “I used to feel pessimistic about the results of my exams. But now, I just perceive exams as a phase test. Therefore, I can do my best to prepare for the exam.” Another participant noted, “I do not want to put off exam-related tasks or avoid exams any longer. It is only right to take action and concentrate on learning!”

All students in the experimental group showed progress in emotional regulation, as evidenced by the following improvements: (a) They were more extroverted. They could share their emotional experience (positive or negative) more easily. They exhibited a more optimistic and positive outlook on life. (b) Their emotional awareness became clearer. They started to understand and accept their negative feelings. They developed better coping strategies for stress and negative emotions. (c) They were better at regulating emotions, and could maintain a better balance between their academic and personal lives. Their goal behavior and execution abilities were improved. Many participants reported a reduction in nervousness and worry related to the exam process and results. They no longer show extreme mood swings, negative emotions, irritability, excessive tension, or panic. One participant remarked, “When facing exams, I am no longer sensitive, easily provoked, irritable, impulsive and can control my emotions. This allowed me to concentrate on the review and improve the revision efficiency.” All these reactions suggest that the enhanced emotional regulation ability can greatly improve test anxiety.

### Examination grade after GIMT

During the four-week follow-up, all participants took the final exam. The GPA of the experimental group was much higher than that of the control group (*p* < 0.01**) (Table 5), indicating the positive effect of the GIMT intervention in alleviating test anxiety.

**Table 5:** Distribution of GPAs of the students in the experimental and control groups.

	Experimental group	Control group	*p*	*t*	95% CI
GPA of last semester	2.20 ± 0.68	2.22 ± 0.65	>0.05	0.10	−0.331 to 0.364
GPA after GIMT	2.41 ± 0.75	1.87 ± 0.71	**	2.85	−0.921 to −0.160

All data were obtained from the corresponding test scales and expressed as mean ± SD. Independent *t*-test was used to assess differences between different groups. CI, confidence interval. ** represents *p* < 0.01.

## Discussion

A lot of studies demonstrate that MT is effective in relieving anxiety (de Witte et al., 2020; Lu et al., 2021; de Witte et al., 2022). As for test anxiety, some MTs were also found to be able to reduce test anxiety (Eyüboğlu et al., 2021; Galal et al., 2021; de Witte et al., 2022). The current study is the first to prove the important and effective role of GIMT in relieving test anxiety. Both the control group and the experimental group exhibited high levels of test anxiety before the intervention, which aligns with the findings of Ugwuanyi et al. (2020) that test anxiety levels tend to rise as exams approach. After the GIMT intervention, the level of test anxiety in the experimental group decreased significantly. This is a positive result indicating the effectiveness of the intervention. Our study also estimated the influence of reduced test anxiety level on academic performance. The experimental group receiving the GIMT intervention achieved a higher average GPA than the control group. These findings are consistent with other studies that links alleviated test anxiety with elevated test scores (Lilley et al., 2014; Gallego-Gómez et al., 2020). Moreover, a 4-week follow-up qualitative study after the GIMT intervention indicated that students in the experimental group were more likely to accept and handle negative emotions, and displayed less anxiety. This qualitative data further supports the quantitative outcomes and provides a deeper understanding of the effect of the intervention on emotional regulation and anxiety levels. Both quantitative analyses and qualitative information demonstrate that the GIMT intervention can effectively improve test anxiety and emotional regulation, suggesting that GIMT might be a promising and valuable tool to help students handle and reduce their test anxiety.

The relationship between test anxiety and emotional regulation has been discussed in many studies. Emotional regulation can reduce anxiety levels during stressful events such as exams (Aldao et al., 2010; Moltrecht et al., 2021). Schäfer et al. suggest that the use of emotional regulation strategies is a key factor in treating anxiety (Schäfer et al., 2017). Therefore, the improvement of test anxiety in the experimental group may be related to the use of regulation strategies. Recently, Liu et al. found that emotional regulation was significantly negatively correlated with test anxiety (Liu et al., 2021), indicating that with the improvement of emotional regulation skills, test anxiety may be reduced. Moreover, emotional regulation has been shown to indirectly affect test anxiety through the mediating effect of psychological resilience (Liu et al., 2021). These findings indicate that it may be possible to reduce the effects of anxiety by helping individuals regulate their emotions. In our study, the emotional regulation ability of the experimental group was significantly improved after the GIMT intervention, as evidenced by the decreased scores of the six dimensions of the DERS. Both the quantitative data and qualitative research also reported that the students who underwent the GIMT intervention exhibited less test anxiety, which may be partially attributed to the improved emotional regulation abilities from the GIMT intervention. All these observations reinforce the potential of emotional regulation in monitoring test anxiety and provide theoretical support for the application of GIMT as an effective therapy to improve emotional regulation skills and thereby reduce test anxiety levels.

There might be mental reasons and biological reasons for the effectiveness of GMIT in alleviating test anxiety. As for the possible mental reasons, we propose two possibilities. One potential mechanism is that music can act as a distractor to diverting the patient’s attention from negative stimuli towards pleasant and encouraging experiences, thus lowering anxiety levels (Bernatzky et al., 2011; Chanda and Levitin, 2013). Another factor is the continuous attunement to patients’ unique needs by the music therapists (Pothoulaki et al., 2012), which create a supportive and empathetic environment. Possible biological reasons for GMIT to improve test anxiety and emotional regulation are as follows. Fachner et al. (2013) found that impromptu MT directly affected brain activity, particularly in the cerebral cortex of depressive patients with comorbid anxiety. The therapy induced frontotemporal nerve reorganization in areas associated with mood regulation, potentially improving depressive and anxiety symptoms. Some Music interventions have been reported to reduce the levels of stress hormones such as *β*-endorphin and cortisol, which are part of the hypothalamic–pituitary–adrenal (HPA) axis (Chanda and Levitin, 2013). The body can release a hormone called adrenaline in response to stresses, including test anxiety (Maduka et al., 2015). Impromptu music may reduce the secretion of adrenaline and noradrenaline (Conrad et al., 2007; Jiménez-Jiménez et al., 2013), therefore alleviating the symptoms of test anxiety. Besides, music listening was shown to be able to activate amygdala (Roozendaal et al., 2009; Nian et al., 2023) and hippocampus (Chanda and Levitin, 2013; Koelsch, 2014; Mao et al., 2022), which are involved in processing emotions or regulating stress. Moreover, music has been found to promote the secretion of dopamine, a neurotransmitter related to pleasure and reward (Ferreri et al., 2021; Speranza et al., 2022) These changes may reduce stress, enhance positive emotional experience, improve impulse control, and finally improve emotional regulation. Therefore, GIMT may also target these psychological and physiological aspects to form a useful method to reduce test anxiety.

Interestingly, there were no gender differences in the effectiveness of the GIMT intervention on test anxiety and emotional regulation in our study. This suggests that GIMT is equally beneficial for both females and males. Traditional socialization practices often encourage males to suppress their emotions, which can lead to increased stress and anxiety (Farhane-Medina et al., 2022). Our study suggests that GIMT might be suitable to help males express positive emotions and manage negative ones, exhibiting high clinical value for male mental health.

Medical students are vulnerable to test anxiety due to the rigorous demands of their education. This anxiety may impair their academic performance and ability to effectively retain and apply knowledge. If not addressed, the stress and anxiety can carry over into their professional lives, potentially affecting the quality of patient care. Simpler and more practical coping strategies that can help medical students recognize and alleviate their stress are needed. These strategies should be accessible and easy to incorporate into their busy schedules. Our study confirmed the effectiveness of GIMT as an intervention for test anxiety and emotional regulation, providing a promising clinical auxiliary method for dealing with test anxiety and an important addition to a healthcare professional school curriculum.

### Limitation

Several limitations of this study should be noted. First, the sample size was relatively small, which can affect the statistical power of a study and the generalizability of its results. Future studies with a larger sample size are needed to provide more reliable results. Second, the results were obtained only from the medical students of a single university, limiting the external validity of the findings. Future research should include a wider range of participants from different medical colleges and potentially from different disciplines. Third, in our experiment, there were more women than men. As there are gender differences in coping with test anxiety, this might introduce bias. We need to strictly pay attention to the gender ratio in future studies. Lastly, there is a lack of blinding process. This may cause participants bias, where participants’ knowledge of the research objective may affect their responses. Double-blind or triple blind study designs in future research will help alleviate this issue. In general, solving these limitations is necessary for the advancement of research GIMT and its clinical application.

## Conclusion

All findings indicate that GIMT, as an intervention tool, plays an important and effective role in relieving test anxiety among medical students. The association between improved emotional regulation ability and reduced test anxiety suggests that GIMT may function by enhancing students’ ability to regulate their emotions. This study investigated the role of GIMT in test anxiety among medical students for the first time, opening up a new research area that can be further refined. The findings provide a distinctive approach that can be integrated into the support systems of medical colleges to help students to cope with academic stress and improve academic development. Overall, the study adds valuable knowledge to the field of mental health interventions in education and highlights the potential of GIMT as a non-pharmacological approach to improving the mental health and academic outcomes of medical students.

## Data Availability

The original contributions presented in the study are included in the article/supplementary material, further inquiries can be directed to the corresponding authors.

## Footnotes

^1^ https://www.randomizer.org/

## References

[ref1] Aldao A. Nolen-Hoeksema S. Schweizer S. (2010). Emotion-regulation strategies across psychopathology: a meta-analytic review. Clin. Psychol. Rev. 30, 217–237. doi: 10.1016/j.cpr.2009.11.004, PMID: 20015584

[ref3] Badrian M. Bazrafkan L. Shakour M. (2022). Medical science students' experiences of test anxiety: a phenomenological study. BMC Psychol. 10:187. doi: 10.1186/s40359-022-00896-4, PMID: 35906665 PMC9336078

[ref4] Baldwin D. S. Anderson I. M. Nutt D. J. Bandelow B. Bond A. Davidson J. R. T.. (2005). Evidence-based guidelines for the pharmacological treatment of anxiety disorders: recommendations from the British Association for Psychopharmacology. J. Psychopharmacol. 19, 567–596. doi: 10.1177/0269881105059253, PMID: 16272179

[ref5] Bandelow B. Werner A. M. Kopp I. Rudolf S. Wiltink J. Beutel M. E. (2022). The German guidelines for the treatment of anxiety disorders: first revision. Eur. Arch. Psychiatry Clin. Neurosci. 272, 571–582. doi: 10.1007/s00406-021-01324-1, PMID: 34609587 PMC8490968

[ref6] Bernatzky G. Presch M. Anderson M. Panksepp J. (2011). Emotional foundations of music as a non-pharmacological pain management tool in modern medicine. Neurosci. Biobehav. Rev. 35, 1989–1999. doi: 10.1016/j.neubiorev.2011.06.005, PMID: 21704068

[ref7] Bodas J. Ollendick T. H. (2005). Test anxiety: a cross-cultural perspective. Clin. Child. Fam. Psychol. Rev. 8, 65–88. doi: 10.1007/s10567-005-2342-x, PMID: 15898305

[ref8] Bolbolian M. Asgari S. Sefidi F. Zadeh A. S. (2021). The relationship between test anxiety and academic procrastination among the dental students. J. Educ. Health Promot. 10:67. doi: 10.4103/jehp.jehp_867_20, PMID: 34084814 PMC8057172

[ref9] Chanda M. L. Levitin D. J. (2013). The neurochemistry of music. Trends Cogn. Sci. 17, 179–193. doi: 10.1016/j.tics.2013.02.007 23541122

[ref10] Chapell M. S. Blanding Z. B. Silverstein M. E. Takahashi M. Newman B. Gubi A.. (2005). Test anxiety and academic performance in undergraduate and graduate students. J. Educ. Psychol. 97, 268–274. doi: 10.1037/0022-0663.97.2.268

[ref11] Conrad C. Niess H. Jauch K. W. Bruns C. J. Hartl W. Welker L. (2007). Overture for growth hormone: requiem for interleukin-6? Crit. Care Med. 35, 2709–2713. doi: 10.1097/00003246-200712000-00005, PMID: 18074473

[ref12] De Witte M. Pinho A. D. S. Stams G. J. Moonen X. Bos A. E. R. Van Hooren S. (2022). Music therapy for stress reduction: a systematic review and meta-analysis. Health Psychol. Rev. 16, 134–159. doi: 10.1080/17437199.2020.1846580, PMID: 33176590

[ref13] De Witte M. Spruit A. Van Hooren S. Moonen X. Stams G. J. (2020). Effects of music interventions on stress-related outcomes: a systematic review and two meta-analyses. Health Psychol. Rev. 14, 294–324. doi: 10.1080/17437199.2019.1627897, PMID: 31167611

[ref14] Dileo C. (2006). Effects of music and music therapy on medical patients: a meta-analysis of the research and implications for the future. J. Soc. Integr. Oncol. 4, 67–70. doi: 10.2310/7200.2006.002 19442338

[ref15] Dowker A. Sheridan H. (2022). Relationships between mathematics performance and attitude to mathematics: influences of gender, test anxiety, and working memory. Front. Psychol. 13:814992. doi: 10.3389/fpsyg.2022.814992, PMID: 35330725 PMC8940274

[ref16] Eyüboğlu G. Göçmen Baykara Z. Çalışkan N. Eyikara E. Doğan N. Aydoğan S.. (2021). Effect of music therapy on nursing students' first objective structured clinical exams, anxiety levels and vital signs: a randomized controlled study. Nurse Educ. Today 97:104687. doi: 10.1016/j.nedt.2020.104687, PMID: 33310698

[ref17] Fachner J. Gold C. Erkkilä J. (2013). Music therapy modulates fronto-temporal activity in rest-EEG in depressed clients. Brain Topogr. 26, 338–354. doi: 10.1007/s10548-012-0254-x, PMID: 22983820

[ref18] Farhane-Medina N. Z. Luque B. Tabernero C. Castillo-Mayén R. (2022). Factors associated with gender and sex differences in anxiety prevalence and comorbidity: a systematic review. Sci. Prog. 105:368504221135469. doi: 10.1177/00368504221135469, PMID: 36373774 PMC10450496

[ref19] Ferreri L. Mas-Herrero E. Cardona G. Zatorre R. J. Antonijoan R. M. Valle M.. (2021). Dopamine modulations of reward-driven music memory consolidation. Ann. N. Y. Acad. Sci. 1502, 85–98. doi: 10.1111/nyas.14656, PMID: 34247392

[ref20] Galal S. Vyas D. Hackett R. K. Rogan E. Nguyen C. (2021). Effectiveness of music interventions to reduce test anxiety in pharmacy students. Pharmacy 9:10. doi: 10.3390/pharmacy9010010 33466485 PMC7838995

[ref21] Gallego-Gómez J. I. Balanza S. Leal-Llopis J. García-Méndez J. A. Oliva-Pérez J. Doménech-Tortosa J.. (2020). Effectiveness of music therapy and progressive muscle relaxation in reducing stress before exams and improving academic performance in nursing students: a randomized trial. Nurse Educ. Today 84:104217. doi: 10.1016/j.nedt.2019.104217, PMID: 31683132

[ref22] Garakani A. Murrough J. W. Freire R. C. Thom R. P. Larkin K. Buono F. D.. (2020). Pharmacotherapy of anxiety disorders: current and emerging treatment options. Front. Psych. 11:595584. doi: 10.3389/fpsyt.2020.595584 PMC7786299 33424664

[ref23] Gomes A. P. Soares A. L. G. Kieling C. Rohde L. A. Gonçalves H. (2019). Mental disorders and suicide risk in emerging adulthood: the 1993 Pelotas birth cohort. Rev. Saude Publica 53:96. doi: 10.11606/s1518-8787.20190530012356, PMID: 31644774 PMC6802944

[ref9001] Guba E. Lincoln Y. (1994). Sage handbook of qualitative research. Sage: Thousand Oaks, CA.

[ref26] Hochberg Z. E. Konner M. (2019). Emerging adulthood, a pre-adult life-history stage. Front. Endocrinol. 10:918. doi: 10.3389/fendo.2019.00918 PMC6970937 31993019

[ref27] Hodo D. W. (2006). Kaplan and Sadock's comprehensive textbook of psychiatry. Am. J. Psychiatry 163:2199.

[ref28] Jerrim J. (2023). Test anxiety: is it associated with performance in high-stakes examinations? Oxf. Rev. Educ. 49, 321–341. doi: 10.1080/03054985.2022.2079616

[ref29] Jiménez-Jiménez M. García-Escalona A. Martín-López A. De Vera-Vera R. De Haro J. (2013). Intraoperative stress and anxiety reduction with music therapy: a controlled randomized clinical trial of efficacy and safety. J. Vasc. Nurs. 31, 101–106. doi: 10.1016/j.jvn.2012.10.002, PMID: 23953858

[ref30] Jirjees F. Odeh M. Al-Haddad A. Ass'ad R. Hassanin Y. Al-Obaidi H.. (2024). Test anxiety and coping strategies among university students an exploratory study in the UAE. Sci. Rep. 14:25835. doi: 10.1038/s41598-024-59739-4, PMID: 39468103 PMC11519940

[ref31] Kallio H. Pietilä A. M. Johnson M. Kangasniemi M. (2016). Systematic methodological review: developing a framework for a qualitative semi-structured interview guide. J. Adv. Nurs. 72, 2954–2965. doi: 10.1111/jan.13031, PMID: 27221824

[ref32] Kaur Khaira M. Raja Gopal R. L. Mohamed Saini S. Md Isa Z. (2023). Interventional strategies to reduce test anxiety among nursing students: a systematic review. Int. J. Environ. Res. Public Health 20:233. doi: 10.3390/ijerph20021233, PMID: 36673999 PMC9858718

[ref33] Koelsch S. (2014). Brain correlates of music-evoked emotions. Nat. Rev. Neurosci. 15, 170–180. doi: 10.1038/nrn3666, PMID: 24552785

[ref34] Kulsoom B. Afsar N. A. (2015). Stress, anxiety, and depression among medical students in a multiethnic setting. Neuropsychiatr. Dis. Treat. 11, 1713–1722. doi: 10.2147/NDT.S83577, PMID: 26213470 PMC4509544

[ref35] Li J. Han Z. R. Gao M. M. Sun X. Ahemaitijiang N. (2018). Psychometric properties of the Chinese version of the difficulties in emotion regulation scale (DERS): factor structure, reliability, and validity. Psychol. Assess. 30, e1–e9. doi: 10.1037/pas0000582, PMID: 29756796

[ref36] Li J. Zhang Y. R. Chan B. S. M. Tan S. N. Lu J. P. Luo X. R.. (2022). Associations between anxiety, depression, and risk of suicidal behaviors in Chinese medical college students. Front. Psych. 13:298. doi: 10.3389/fpsyt.2022.1012298 PMC9757065 36532186

[ref37] Lilley J. L. Oberle C. D. Thompson J. G. Jr. (2014). Effects of music and grade consequences on test anxiety and performance. Educ. Pub. Foundat. 24, 184–190. doi: 10.1037/pmu0000038

[ref38] Lim L. Chan H. N. Chew P. H. Chua S. M. Ho C. Kwek S. K. D.. (2015). Ministry of Health clinical practice guidelines: anxiety disorders. Singapore Med. J. 56, 310–316. doi: 10.11622/smedj.2015088, PMID: 26106237 PMC4469848

[ref39] Liu Y. Pan H. Yang R. Wang X. Rao J. Zhang X.. (2021). The relationship between test anxiety and emotion regulation: the mediating effect of psychological resilience. Ann. General Psychiatry 20:40. doi: 10.1186/s12991-021-00360-4, PMID: 34488816 PMC8419945

[ref40] Lu G. Jia R. Liang D. Yu J. Wu Z. Chen C. (2021). Effects of music therapy on anxiety: a meta-analysis of randomized controlled trials. Psychiatry Res. 304:114137. doi: 10.1016/j.psychres.2021.114137 34365216

[ref41] Luo L. Yuan J. Bi S. Wang Y. Cao Y. Wen S.. (2024). The impact of emerging adulthood characteristics on college students’ anxiety: the mediating role of life satisfaction and internet addiction. Curr. Psychol. 43, 17332–17342. doi: 10.1007/s12144-024-05615-3

[ref42] Maduka I. C. Neboh E. E. Ufelle S. A. (2015). The relationship between serum cortisol, adrenaline, blood glucose and lipid profile of undergraduate students under examination stress. Afr. Health Sci. 15, 131–136. doi: 10.4314/ahs.v15i1.18, PMID: 25834541 PMC4370130

[ref43] Mao X. Cai D. Lou W. (2022). Music alleviates pain perception in depression mouse models by promoting the release of glutamate in the hippocampus of mice to act on GRIK5. Nucleosides Nucleotides Nucleic Acids 41, 463–473. doi: 10.1080/15257770.2022.2051048, PMID: 35357273

[ref44] Mao Y. Zhang N. Liu J. L. Zhu B. He R. X. Wang X. (2019). A systematic review of depression and anxiety in medical students in China. BMC Med. Educ. 19:327. doi: 10.1186/s12909-019-1744-2, PMID: 31477124 PMC6721355

[ref45] Mccormick S. Lamberson J. (2024). Interventions for test anxiety in nursing students: a literature review. Teach. Learn. Nurs. 19, e404–e411. doi: 10.1016/j.teln.2024.01.005

[ref46] Mcrae K. Gross J. J. (2020). Emotion regulation. Emotion 20, 1–9. doi: 10.1037/emo0000703 31961170

[ref47] Mcvey A. J. Schiltz H. K. Coffman M. Antezana L. Magnus B. (2022). A preliminary psychometric analysis of the difficulties with emotion regulation scale (DERS) among autistic adolescents and adults: factor structure, reliability, and validity. J. Autism Dev. Disord. 52, 1169–1188. doi: 10.1007/s10803-021-05018-4, PMID: 33886035

[ref48] Moltrecht B. Deighton J. Patalay P. Edbrooke-Childs J. (2021). Effectiveness of current psychological interventions to improve emotion regulation in youth: a meta-analysis. Eur. Child Adolesc. Psychiatry 30, 829–848. doi: 10.1007/s00787-020-01498-4, PMID: 32108914 PMC8140974

[ref49] Nazir M. A. Izhar F. Talal A. Sohail Z. B. Majeed A. Almas K. (2021). A quantitative study of test anxiety and its influencing factors among medical and dental students. J. Taibah Univ. Med. Sci. 16, 253–259. doi: 10.1016/j.jtumed.2020.12.014, PMID: 33897331 PMC8046942

[ref50] Neil A. L. Christensen H. (2009). Efficacy and effectiveness of school-based prevention and early intervention programs for anxiety. Clin. Psychol. Rev. 29, 208–215. doi: 10.1016/j.cpr.2009.01.002, PMID: 19232805

[ref51] Nian H. Ding S. Feng Y. Liu H. Li J. Li X.. (2023). Effect of noise and music on neurotransmitters in the amygdala: the role auditory stimuli play in emotion regulation. Meta 13:928. doi: 10.3390/metabo13080928 PMC10456833 37623873

[ref52] Pothoulaki M. Macdonald R. Flowers P. (2012). An interpretative phenomenological analysis of an improvisational music therapy program for cancer patients. J. Music. Ther. 49, 45–67. doi: 10.1093/jmt/49.1.45, PMID: 22803257

[ref53] Putwain D. W. Von Der Embse N. P. Rainbird E. C. West G. (2021). The development and validation of a new multidimensional test anxiety scale (MTAS). Eur. J. Psychol. Assess. 37, 236–246. doi: 10.1027/1015-5759/a000604

[ref54] Quinn B. L. Peters A. (2017). Strategies to reduce nursing student test anxiety: a literature review. J. Nurs. Educ. 56, 145–151. doi: 10.3928/01484834-20170222-05, PMID: 28263352

[ref55] Roozendaal B. Mcewen B. S. Chattarji S. (2009). Stress, memory and the amygdala. Nat. Rev. Neurosci. 10, 423–433. doi: 10.1038/nrn2651 19469026

[ref56] Sandak B. Cohen S. Gilboa A. Harel D. (2019). Computational elucidation of the effects induced by music making. PLoS One 14:e0213247. doi: 10.1371/journal.pone.0213247, PMID: 30845183 PMC6405055

[ref57] Schäfer J. Naumann E. Holmes E. A. Tuschen-Caffier B. Samson A. C. (2017). Emotion regulation strategies in depressive and anxiety symptoms in youth: a meta-analytic review. J. Youth Adolesc. 46, 261–276. doi: 10.1007/s10964-016-0585-0, PMID: 27734198

[ref58] Shao R. Y. He P. Ling B. Tan L. Xu L. Hou Y. H.. (2020). Prevalence of depression and anxiety and correlations between depression, anxiety, family functioning, social support and coping styles among Chinese medical students. BMC Psychol. 8:38. doi: 10.1186/s40359-020-00402-8 32321593 PMC7178943

[ref59] Shi M. Lu X. S. Du T. J. (2022). Associations of trait emotional intelligence and stress with anxiety in Chinese medical students. PLoS One 17:e0273950. doi: 10.1371/journal.pone.0273950, PMID: 36048865 PMC9436114

[ref60] Speranza L. Pulcrano S. Perrone-Capano C. Di Porzio U. Volpicelli F. (2022). Music affects functional brain connectivity and is effective in the treatment of neurological disorders. Rev. Neurosci. 33, 789–801. doi: 10.1515/revneuro-2021-0135, PMID: 35325516

[ref61] Ugwuanyi C. S. Ede M. O. Onyishi C. N. Ossai O. V. Nwokenna E. N. Obikwelu L. C.. (2020). Effect of cognitive-behavioral therapy with music therapy in reducing physics test anxiety among students as measured by generalized test anxiety scale. Medicine 99:e16406. doi: 10.1097/MD.0000000000016406, PMID: 32332590 PMC7220727

[ref62] Von Der Embse N. Jester D. Roy D. Post J. (2018). Test anxiety effects, predictors, and correlates: a 30-year meta-analytic review. J. Affect. Disord. 227, 483–493. doi: 10.1016/j.jad.2017.11.048, PMID: 29156362

[ref63] Wang C. K. (2001). Reliability and Validity of Test Anxiety Scale-Chinese Version. Chin. Mental Health J. 15, 96–97.

[ref64] Yusoff M. S. B. Abdul Rahim A. F. Baba A. A. Ismail S. B. Mat Pa M. N. Esa A. R. (2013). Prevalence and associated factors of stress, anxiety and depression among prospective medical students. Asian J. Psychiatr. 6, 128–133. doi: 10.1016/j.ajp.2012.09.012, PMID: 23466109

[ref65] Zeng W. Chen R. Q. Wang X. Y. Zhang Q. Deng W. (2019). Prevalence of mental health problems among medical students in China a meta-analysis. Medicine 98:e15337. doi: 10.1097/MD.0000000000015337, PMID: 31045774 PMC6504335

[ref9002] Zhang M. Ding Y. Zhang J. Jiang X. Xu N. Zhang L. (2022). Effect of Group Impromptu Music Therapy on Emotional Regulation and Depressive Symptoms of College Students: A Randomized Controlled Study. Front Psychol 13:851526. doi: 10.3389/fpsyg.2022.851526 35432107 PMC9008882

